# Systems mapping of multilevel factors contributing to dental caries in adolescents

**DOI:** 10.3389/froh.2023.1285347

**Published:** 2024-01-31

**Authors:** Fatima Sadjadpour, Niyousha Hosseinichimeh, Bhavna T. Pahel, Sara S. Metcalf

**Affiliations:** ^1^ Department of Industrial and Systems Engineering, Virginia Polytechnic Institute and State University, Falls Church, VA, United States; ^2^ Private Practice of Pediatric Dentistry in Easley and Anderson, Easley, SC, United States; ^3^ Department of Geography, The State University of New York at Buffalo, Buffalo, NY, United States

**Keywords:** dental caries, adolescents, systems science, system dynamics, causal loop diagram

## Abstract

Dental caries is a prevalent chronic disease among adolescents. Caries activity increases significantly during adolescence due to an increase in susceptible tooth surfaces, immature permanent tooth enamel, independence in pursuing self-care, and a tendency toward poor diet and oral hygiene. Dental caries in permanent teeth is more prevalent among adolescents in low-income families and racial/ethnic minority groups, and these disparities in adolescent dental caries experience have persisted for decades. Several conceptual and data-driven models have proposed unidirectional mechanisms that contribute to the extant disparities in adolescent dental caries experience. Our objective, using a literature review, is to provide an overview of risk factors contributing to adolescent dental caries. Specifically, we map the interactive relationships of multilevel factors that influence dental caries among adolescents. Such interactive multilevel relationships more closely reflect the complex nature of dental caries experience among the adolescent population. The methods that we use are two-fold: (1) a literature review using PubMed and Cochrane databases to find contributing factors; and (2) the system dynamics approach for mapping feedback mechanisms underlying adolescent dental caries through causal loop diagramming. The results of this study, based on the review of 138 articles, identified individual, family and community-level factors and their interactions contributing to dental caries experience in adolescents. Our results also provide hypotheses about the mechanisms underlying persistence of dental caries among adolescents.

**Conclusions:**

Our findings may contribute to a deeper understanding of the multilevel and interconnected factors that shape the persistence of dental caries experience among adolescents.

## 1 Introduction

Dental caries is a complex, costly, and prevalent chronic disease. It is affected by multi-level factors ranging from individual's salivary characteristics (1), fluoride exposure (2) and dental anxiety (3), to family's health literacy (4), socioeconomic status (5) and community's geographical context and water fluoridation status (6). Rather than acting separately, these multilevel factors often interact with each other. According to the World Health Organization, dental caries is the 4th most expensive disease to treat (7). In the United States, 58% of adolescents aged 12–19 years have experienced dental caries from 2011 through 2014. The prevalence of dental caries is even higher (i.e., 66%) for adolescents in families whose income is less than 100% of the Federal Poverty Level (FPL) (8). The disparity in dental caries of adolescents’ experience has been persistent, as evidenced by lack of improvement relative to younger age cohorts This issue is not exclusive to the United States; a similar trend is observable globally (9). For instance, the National Australian Child Dental Survey conducted in 2003–2004 reported that 40%–57% of adolescents aged 12–15 had experienced dental caries (10), which highlights the widespread nature of this complex disease.

Fisher-Owens et al. (11) conducted a comprehensive study of factors, using the ecological approach, to depict a conceptual model of multilevel factors influencing children's oral health, including genetic and biological factors, the social environment, the physical environment, health behaviors, and dental and medical care. Although the authors acknowledged the presence of causal relationships and feedback loops, to the best of our knowledge, no study has hypothesized and mapped the causal reciprocal relationships among multilevel factors that influence dental caries among adolescents.

Multi-level factors that influence adolescents’ dental caries are not isolated and often interact, which makes reducing disparities in dental caries a challenging task. Systems science complements other analytic techniques by accounting for interactions among factors, delays in the system, and feedback mechanisms (12). In this study, we conduct a literature review of adolescents’ dental caries experience to identify the reciprocity of multi-level factors contributing to this chronic disease. We further categorize these contributing factors and present their frequency to provide an overview of the literature and highlight areas of opportunities for future interventions to reduce disparities in adolescents’ dental caries experience. We also apply a qualitative system dynamics approach (13) to hypothesize and map the reciprocal relationships of the identified factors. The strength of empirical support for each causal mechanism was assessed and prioritized for those that had been validated through the literature.

## 2 Methods

### 2.1 Search strategy

We conducted a literature review to identify journal publications related to adolescents’ dental caries experience. We conducted our search using two databases, PubMed and Cochrane library, between 1979 and 2021. The following keywords were searched in the title and abstract of both databases: dental caries and adolescent (PubMed: (dental caries [Title/Abstract]) AND (adolescent [Title/Abstract]), Cochrane library: (dental caries [Title/Abstract]) AND (adolescent [Title/Abstract]) with Oral Health, Child Health in Cochrane groups).

### 2.2 Inclusion and exclusion criteria

**Figure 1:**
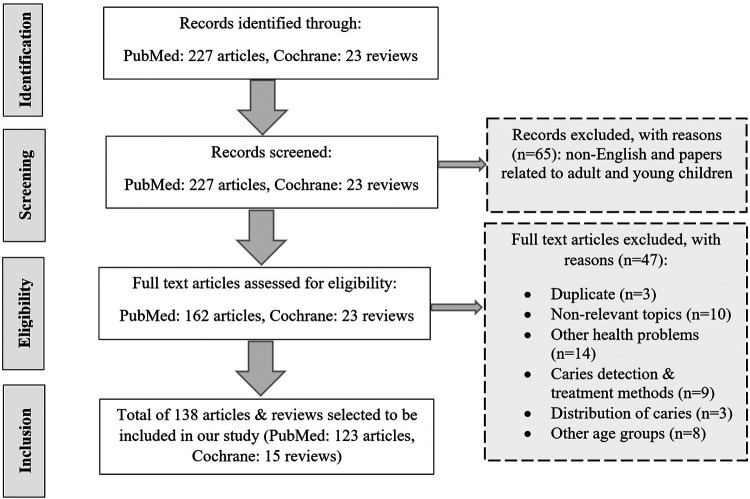
Flow diagram of the literature review process.

After an initial screening of the papers found in PubMed, we excluded articles not written in the English language and articles related to adults and young children. Also, after consulting with specialists in the field of oral health, we decided to only include Cochrane reviews and exclude Cochrane protocols and trials from the search results found in the Cochrane library. Then we combined all the articles found from the two databases and excluded the duplicates. After reading the abstracts of our combined list of papers, we excluded articles that focused on non-relevant topics, other health problems, comparison of caries detection & treatment methods, the distribution of caries among various teeth, and other age groups (Figure 1).

### 2.3 Study selection

After screening the abstract of the papers found in PubMed and Cochrane library, we selected 138 papers to be included in our study. From reviewing the selected papers, we identified factors affecting dental caries in adolescents that were studied in these papers and categorized the papers according to these identified factors. Then we mapped the feedback mechanisms that were revealed from this review of the literature using the Vensim software (https://vensim.com/software/). Key steps and results of this process are summarized below, with additional details provided in the Supplementary Material.

## 3 Results

### 3.1 Identification of factors

**Figure 2:**
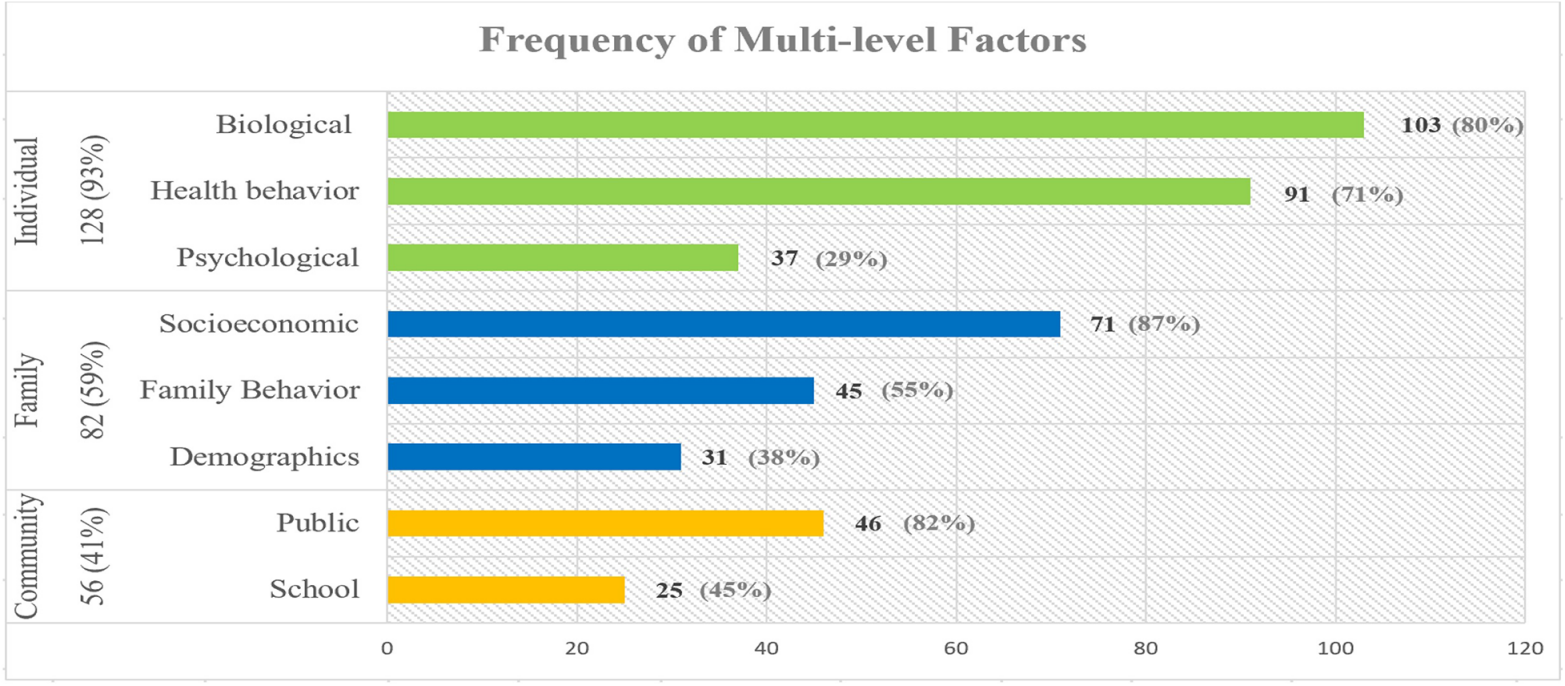
Frequency of multi-level factors.

After reviewing the 138 articles selected by our search strategy, we identified 8 factors at 3 levels: individual level (*n* = 128), family level (*n* = 82), and community level (*n* = 56). Figure 2 demonstrates the categorization of articles by level along with the number of articles for each factor. Individual-level studies focused on three types of factors: biological (*n* = 103), health behavior (*n* = 91) and psychological (*n* = 37). Family-level articles focused on three types of factors: family socioeconomic status (*n* = 71), family behavior (*n* = 45) and family demographics (*n* = 31) factors. Community-level articles focused on two factors: public (*n* = 46) and school (*n* = 25). In the following sections, we further subcategorize each of these factors and summarize the related articles.

#### 3.1.1 Individual-level factors

**Figure 3:**
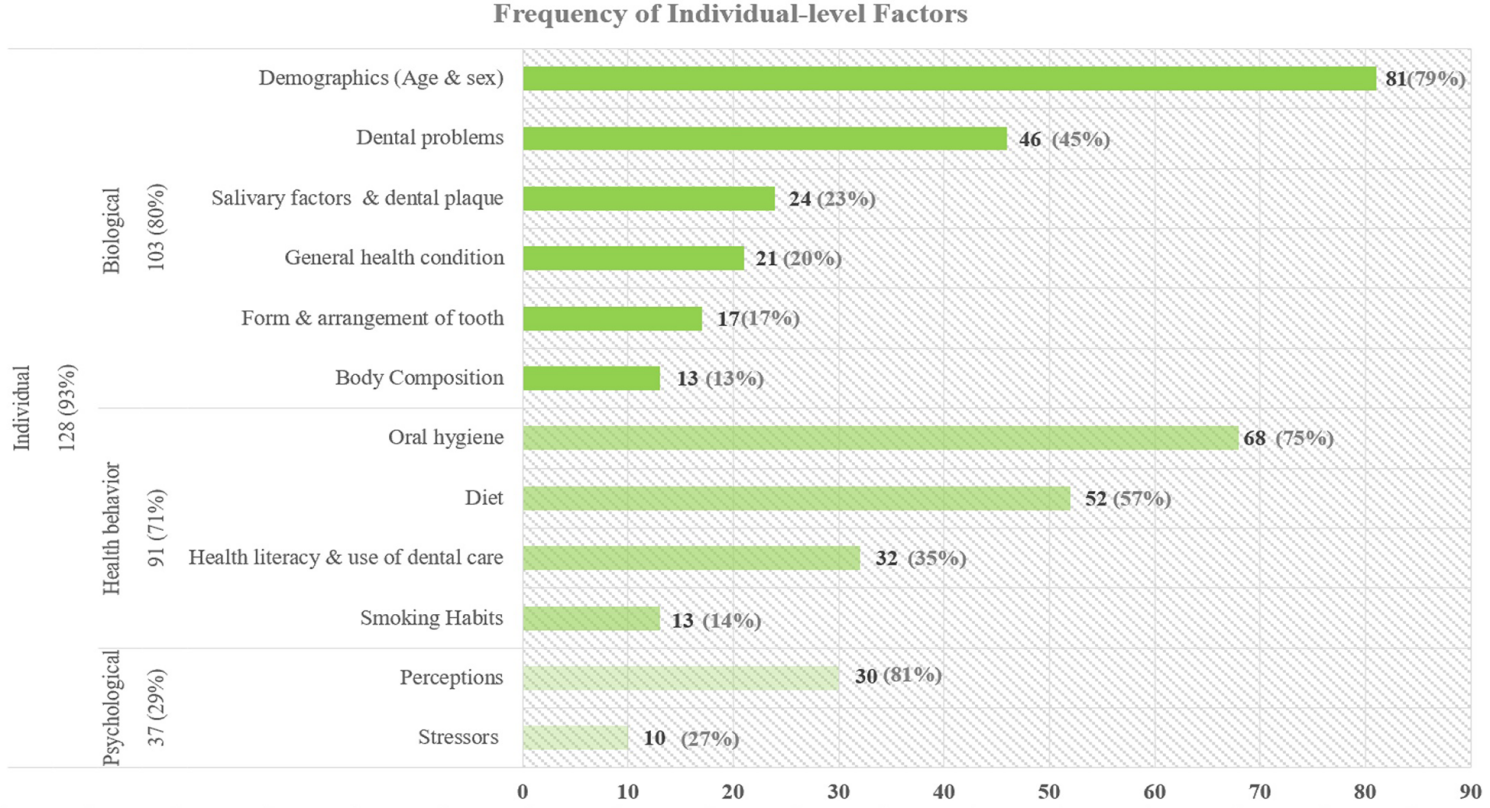
Frequency of individual level factors.

The most common factors studied in dental caries research are at the individual level. Figure 3 depicts the frequency of each factor and related subcategories in individual-level articles.

##### 3.1.1.1 Biological

Biological factors are among the most common influences being studied in the research related to adolescent dental caries. Most studies controlled for demographics of subjects such as age and sex, and some found that adolescent girls have a higher chance of developing caries (14–17). Among dental problems, gingival conditions such as gingival bleeding and periodontal gum disease are examined in some caries research, as they both often result from poor oral hygiene that also leads to dental caries (18–20). Traumatic dental injuries and tooth pain, resulting from dental caries (21), have a negative impact on the oral health status of adolescents (22, 23). Studies show a positive association among caries index and visible plaque or dental biofilm (24), high levels of S. mutans and lactobacilli (25), salivary flow rate, reduced pH and low buffering capacity (26–28). In addition, genetic disorders such as Down syndrome (29), cleft syndrome (30), and family history of dental caries (31) were investigated. Multiple studies in the literature have analyzed the relationships between dental caries and several health conditions such as diabetes and poor metabolic control (15, 19, 32), obesity (33–35), juvenile idiopathic arthritis (36), and asthma (37, 38). Malocclusion is one of the conditions in form and arrangement of tooth, which is an identified risk factor in dental caries research as alignment and spacing of early permanent dentition is an important factor in developing caries (39–43). In addition, enamel defects in low birth weight adolescents (which are detectable once the tooth has emerged into the oral cavity) (44), body mass index (BMI), and waist circumference from body composition factors are significantly associated with dental caries (45).

##### 3.1.1.2 Health behavior

Multiple components of adolescent health behavior including oral hygiene, dietary habits, and use of dental care have been studied widely. Oral hygiene (43, 46), specifically toothbrushing twice a day (43, 47, 48) using a fluoridated toothpaste, is consistently found to reduce dental caries (49–52). In addition, use of dental floss (53) and topical fluoride exposure (54, 55), such as fluoride mouth rinse (56), fluoride gels (57) and fluoride varnishes (58) are also recommended as their benefits for oral health are well established throughout the literature. The other health behavior frequently mentioned in studies of dental caries is diet (59). Several studies have shown that sugar consumption (33, 60–63), specifically sugar before bedtime (64), frequent snacking (46, 65, 66), irregular main meals, and skipping breakfast (65, 67) increase the risk of developing caries among adolescents. Health literacy and use of dental care affect dental caries (48, 68). Health literacy is positively associated with use of dental care (48, 69), communication with a dentist (48) and healthy diet (70), which enhance oral health quality of life (18). In addition, use of pit and fissure sealants prevents dental caries in adolescents (24, 54, 71–73). On the other hand, dental caries and oral health status of adolescents may affect school performance (74), school absenteeism (21), and future employment outcome (75), and consequently health literacy and use of dental care. Finally, other habits, such as smoking (35, 60, 65, 66) and use of snuff or smokeless tobacco (60), are also risk factors, as tobacco exposure (76) is associated with dental caries among adolescents. This association might work in both directions, as one study suggests that having dental caries in adolescence could be an indicator of becoming a smoker in adulthood (77). Alcohol intake has also been evaluated as a risk factor in some dental caries research (35, 60, 78, 79).

##### 3.1.1.3 Psychological

Increasing evidence also highlights the impact of self-perception on adolescents’ dental caries experience and oral health status. Having healthy teeth is socially more desirable and increases adolescent's happiness (42), while untreated dental caries can negatively impact adolescents’ self-esteem and oral health quality of life (68, 80, 81). Adolescents are susceptible to peer influence which affects their health behavior and diet (81). Self-efficacy seems to play an important role in improving oral health practices through diet modification, reduction of sugar consumption, frequent toothbrushing and dental flossing (53, 81–84). High sense of coherence is usually associated with good health behaviors and studies indicate that adolescents with lower sense of coherence (85) are more likely to develop dental caries. On the other hand, another study has shown a positive association between higher internal locus of control (those who believe internal factors are responsible for their health) and dental caries risk (86). Avoidance behavior and unmet dental care needs of adolescents can be due to some common stressors such as fear of pain and dental anxiety (87), which may result from traumatic events and repeated painful experiences (88). Multiple studies have shown that dental caries prevalence is higher among adolescents who have higher levels of dental anxiety (18, 53, 89, 90).

#### 3.1.2 Family-level factors

**Figure 4:**
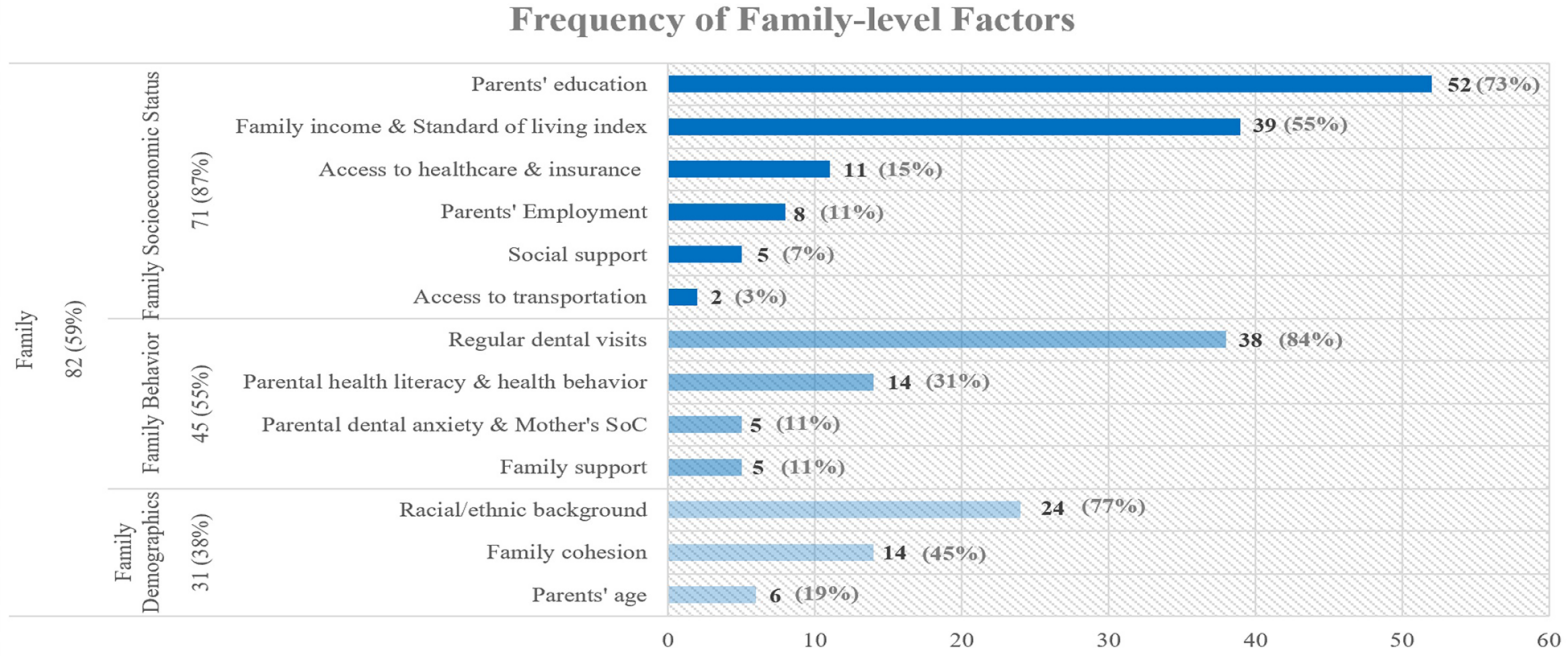
Frequency of family level factors.

The second most common factors in literature were identified at the family level. Figure 4 presents the frequency of family level subfactors discussed in the articles.

##### 3.1.2.1 Family socioeconomic

Several socioeconomic factors can contribute to untreated dental caries among adolescents, such as parents’ education, especially the mother's as the usual active caregiver, which can influence family and adolescents’ health literacy (16, 48, 84, 91, 92), family income, which can impact the household's access to dental care and ability to maintain a healthy diet (27, 92–95), access to healthcare and health insurance (14, 59, 96, 97), parents’ employment, especially the father's, which is usually how their children get dental insurance and can affect adolescents’ health behavior (84), access to social support and social benefits such as unemployment insurance (79, 81, 94, 98) and lack of access to transportation (43, 54).

##### 3.1.2.2 Family behavior

Regular dental visits protect against adolescent dental caries, but their occurrence depends on parental commitment (47, 48, 66, 68, 69, 91, 99). Parental health literacy, especially mother's oral health knowledge (48, 54, 91) is negatively associated with adolescent's dental caries experience. Also, health values, beliefs, lifestyle (27, 43, 54, 96) and health behavior of parents (4, 81, 100) are major risk factors for adolescent dental caries prevalence. According to multiple studies, mothers’ sense of coherence (SoC) has a protective effect against adolescents’ dental caries (85, 101, 102). Also, parental dental anxiety, in terms of experiencing stress and painful dental treatment seems to impact their attitude toward seeking dental care for their children (96), which can consequently increase adolescent's chance of developing caries (91). Family support meaning having parents who promote and supervise good oral health, positively impacts oral health status of adolescents (43, 81, 85), while parental punishment is associated with high levels of caries among adolescents (103).

##### 3.1.2.3 Family demographics

In addition, racial/ethnic background such as belonging to a non-white ethnicity or minority groups (5, 14, 48, 62, 69, 96) and being a recent immigrant (27) increase odds of dental caries. Also, higher level of family cohesion has a positive impact on the oral health literacy and dental visits of adolescents, which will consequently reduce their chances of developing caries (48, 69). Having separated parents and higher number of residents in the household are positively associated with untreated dental caries (94). Studies have included variables related to parental age (4, 15, 44, 48, 69, 91) such as young parents with less education in their analysis as a possible risk factor for adolescents’ dental caries.

#### 3.1.3 Community-level factors

**Figure 5:**
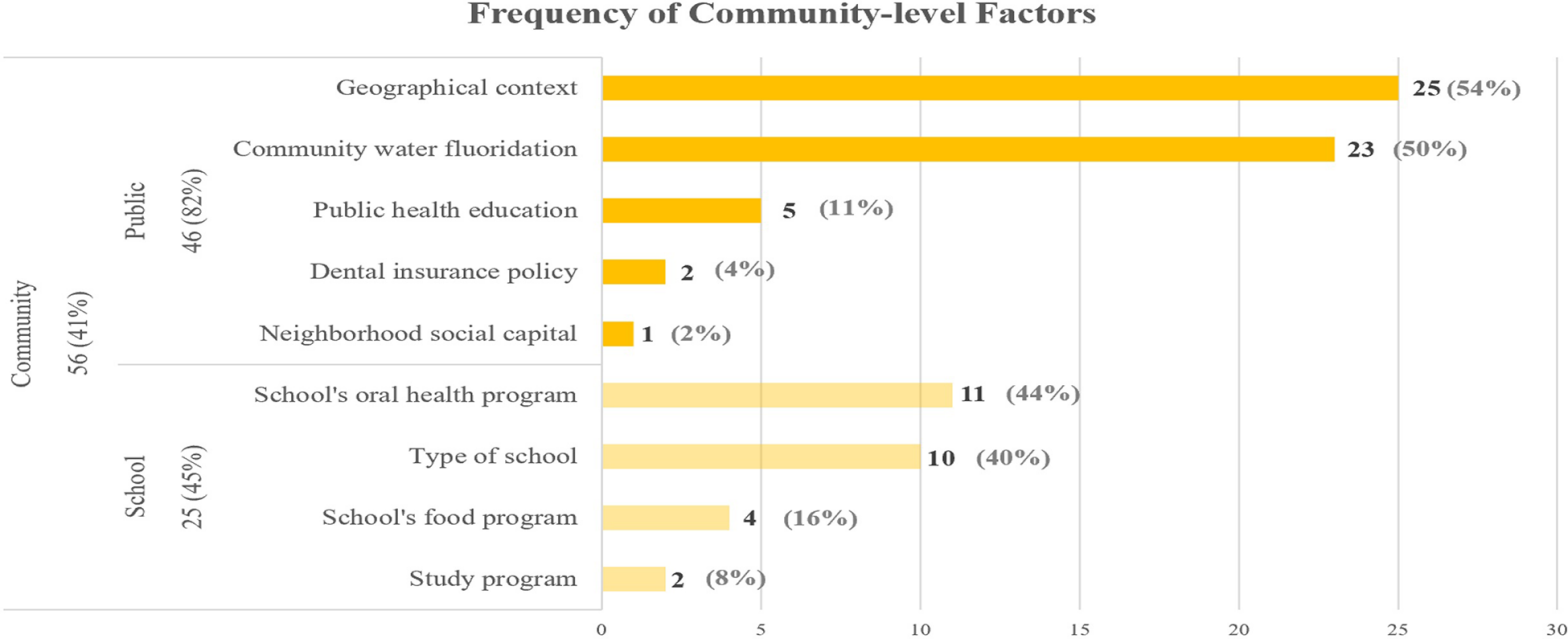
Frequency of community level factors.

The third most common factors in this literature review were identified at the community level. Figure 5 shows the frequency of community level subfactors among articles.

##### 3.1.3.1 Public

Most studies that examine the effect of geographical context on adolescents’ chance of developing caries, have found that living in a rural or remote area is associated with higher rates of dental caries (10, 17, 103, 104), while one study in Sweden showed that adolescents living in urban areas had a higher prevalence of dental caries, possibly due to higher access to cariogenic diet (105). Community water fluoridation is one of the most important community level factors, with its anti-caries benefit being well established in the literature (6, 10, 14, 47, 106–109). Previous studies have emphasized the importance of public health education in the form of educating the community about oral health (43), promotion of oral hygiene at maternal and child healthcare centers (97), preventive dental programs (110), dental health education (111) and interventions for reducing sugar consumption (112). Dental insurance policies such as low reimbursement by Medicaid (43) and coverage expansion of dental care (93) affect adolescents’ dental health. One study had included neighborhood social capital (which measures the trust and norms of reciprocity in the society) for analyzing inequalities in dental caries among adolescents but didn’t find any significant association between the two (79).

##### 3.1.3.2 School

Prevalence of dental caries among adolescents seems to be dependent on several school related factors including school based oral health programs that may include dental screenings, oral health education and supervised fluoride mouth rinse programs (56, 97, 113–116), type of school (for example, higher prevalence of dental caries reported in public schools than private schools) (27, 68, 86, 92, 104), school's food program such as availability of healthy meals, drinking water and fruits instead of vending machines full of sugary drinks and snacks (43, 54, 112, 117), and finally adolescents’ study program (general, sports, vocational), which entails a particular social context that leads them to choose that program (for example, lower prevalence of dental caries for those enrolled in general studies and sports vs. the vocational program) (66, 78).

Results of this literature review demonstrate that among 138 selected articles, about 34% (*n* = 47) of the papers have only focused on one level, while about 40% (*n* = 54) papers have focused on two levels and only about 26% (*n* = 37) of the papers have focused on all three levels. This result shows that few studies have examined diverse factors in adolescents’ dental research (lack of detail complexity). In addition, dynamic complexity of adolescents’ dental caries, which arises from interactions among multiple factors, have not been explored in the literature. In the following section, we present the first map of reciprocal influences which is built upon our literature review.

### 3.2 Mapping feedback mechanisms

**Figure 6:**
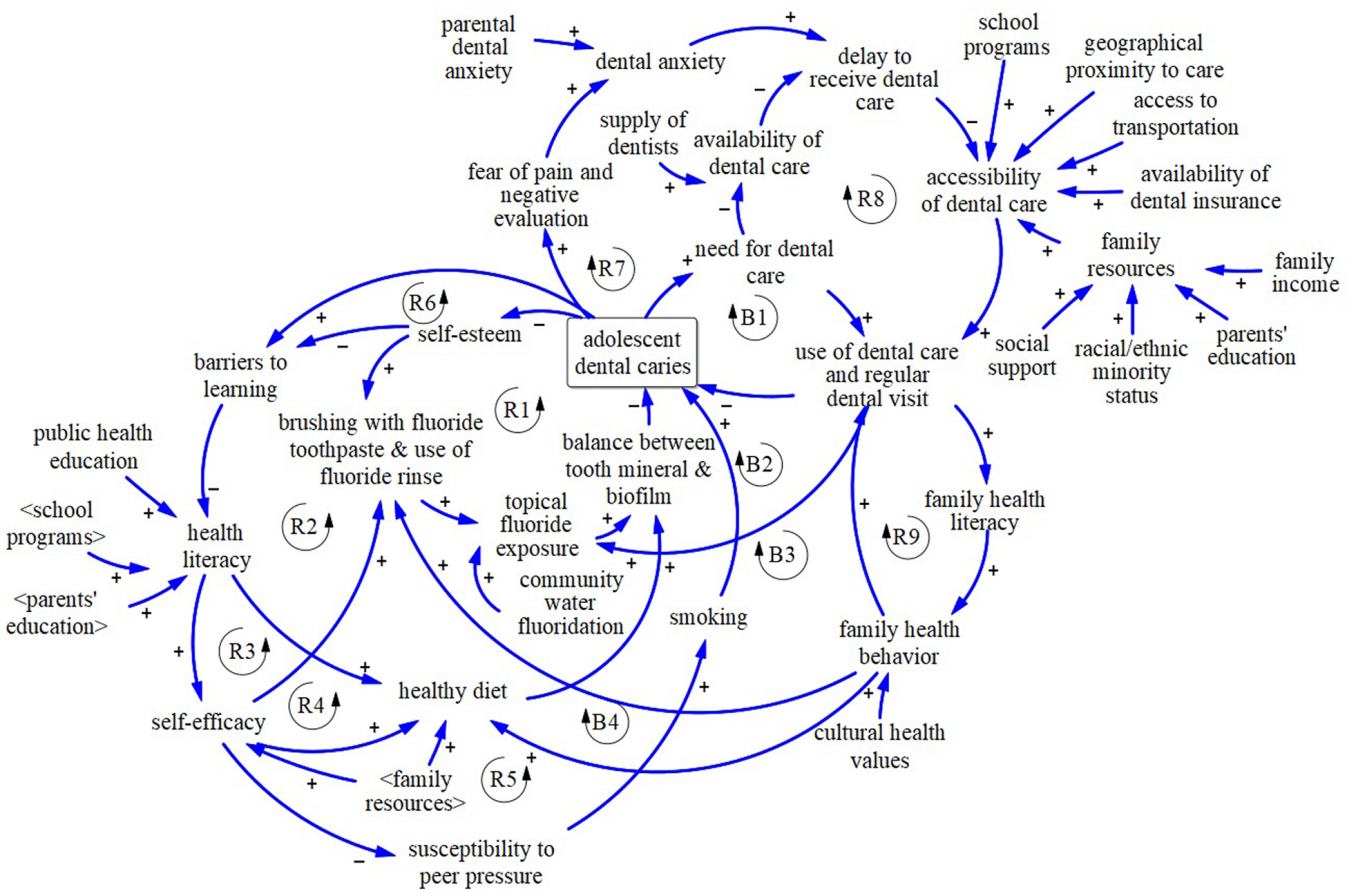
Causal loop diagram of feedback mechanisms affecting adolescents’ dental caries. 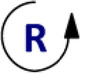: Reinforcing Loop. R1: Dental caries & Deterioration in Self-esteem. R2: Learning Barriers, Health Literacy & Diet. R3: Health Literacy & Self-efficacy. R4: Self-efficacy & Diet. R5: Self-efficacy & Peer Pressure. R6: Dental caries & Barriers (School Performance). R7: Fear of Pain & Dental Anxiety. R8: Overburdened Dental clinics & Treatment Delay. R9: Family Health Behavior & Regular Dental Visits. 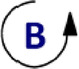: Balancing Loop. B1: Need-Based Dental Visit. B2: Dental Visit & Fluoride Exposure. B3: Dental Visit & Family Health Behavior. B4: Family Health Behavior & Healthy Diet.

System dynamics is an approach for understanding the structure of complex systems and analyzing their behaviors (118–120). Dynamic complexities arise from interactions between elements of a system expressed as feedback mechanisms and accumulations of people, materials or information. A feedback loop is a series of variables and causal links that create a closed loop of causal influences (Figure 6). An arrow with a positive sign means that a change in the first variable produces a change in the second variable in the same direction, keeping all else constant. For example, the arrow relating two variables in Figure 6, “brushing with fluoride toothpaste” and “topical fluoride exposure” is positive, which means that increase in the former leads to an increase in the latter. An arrow with a negative sign means that a change in the first variable produces a change in the second variable in the opposite direction, keeping all else constant. For example, as shown in Figure 6, an increase in adolescent dental caries lowers self-esteem.

All dynamics are created from the interaction of two types of feedback mechanisms, expressed in reinforcing loops and balancing loops. Reinforcing (positive) feedback loops amplify the direction of original movement of any variable in the loop. For instance, reinforcing loop R1 in Figure 6, depicts that higher self-esteem improves oral hygiene behaviors such as brushing with fluoride toothpaste (121, 122) and use of fluoride rinse (123), which increases topical fluoride exposure. Higher topical fluoride exposure improves balance between mineral and biofilm, which lowers dental caries experience and leads to even higher self-esteem (124). Balancing (negative) feedback loops counteract the direction of the original change of a variable in the loop. For example, after experiencing dental caries, one may perceive the need for dental care and then may use it, which would reduce dental caries experience (balancing loop B1 in Figure 6). A causal loop diagram, which consists of reinforcing and balancing feedback loops, is used to show the reciprocal relationships among variables of a system (Figure 6).

### 3.3 Causal loop diagram

After identifying factors influencing adolescent dental caries in the literature, we mapped their reciprocal relationships using the system dynamics approach (Figure 6). Table 1 presents references for the factors included in the causal loop diagram that are extracted from the literature. A brief description of the causal loop diagram follows.

**Table 1:** Multi-level factors in the causal loop diagram that are extracted from the literature.

Factors in the feedback loops	Associated level	References
Reinforcing loop R1		
Self-esteem	Individual	(43, 68, 78, 80, 81, 125–127)
Brushing with fluoride	Individual	(16–19, 27, 28, 31, 33, 35, 38, 43, 44, 46–48, 50, 53, 57, 59, 60, 62–66, 73, 78, 79, 81, 84, 85, 92, 93, 97, 99, 102, 103, 107, 114, 115, 128–134)
Topical fluoride exposure	Individual	(15–17, 24, 27, 31, 43, 44, 46, 47, 49–52, 54, 55, 58, 72, 73, 92, 97, 99, 103, 107, 111, 115, 117, 129, 56, 130, 135)
Balance between tooth mineral and biofilm (visible plaque)	Individual	(15, 19, 24, 31, 33, 34, 37, 38, 43, 59, 73, 101, 102, 111, 115)
Reinforcing loop R2, R3, R4, R6		
Barriers to learning (School performance, absenteeism)	Individual	(21, 67, 74, 75, 102, 112, 125, 136)
Health literacy	Individual	(17, 18, 41, 68–70, 81, 115, 135)
Healthy diet	Individual	(16, 17, 24, 27, 43, 48, 54, 59, 67, 73, 78, 79, 81, 111, 132, 134)
Self-efficacy	Individual	(53, 81–84)
Reinforcing loop R5		
Susceptibility to peer pressure	Individual	(92, 137, 138)
Smoking	Individual	(17, 34, 35, 60, 65, 66, 78, 79)
Reinforcing loop R7		
Fear of pain and negative evaluation	Individual	(87, 88)
Dental anxiety	Individual	(18, 53, 87–90)
Delay to receive dental care	Family	–
Accessibility to dental care	Family	(43, 59, 67, 91, 96, 97, 139)
Use of dental care and Regular dental visit	Family	(19, 24, 27, 31, 35, 42, 47, 48, 53, 54, 62, 66, 68, 69, 71–73, 79, 85, 90–93, 96, 97, 99, 102, 140)
Reinforcing loop R8, Balancing loop B1, B2		
Need for dental care (Due to tooth pain)	Individual	(10, 20, 21, 42, 47, 62, 69, 74, 75, 97, 98, 130, 135, 136, 141)
Availability of dental care	Community	–
Reinforcing loop R9, Balancing loop B3, B4		
Family health literacy	Family	(4, 27, 48, 54, 91, 97)
Family health behavior	Family	(4, 43, 67, 81, 87, 91, 100, 102)
Exogenous factors		
Geographical proximity to care	Community	(4–6, 10, 17, 21, 32, 35, 41, 43, 56, 62, 66, 67, 79, 97, 99, 103–105, 108, 114, 135, 136, 141)
School programs	Community	(17, 21, 43, 54, 81, 97, 112–117, 56, 57)
Parents’ education	Family	(5, 15, 16, 18–20, 22, 28, 33, 39, 40, 42–45, 47, 48, 53, 59, 60, 62, 63, 66, 67, 69, 75, 78, 84, 85, 91, 92, 95, 98, 99, 103, 104, 107, 114, 130, 131, 134, 136)
Family income	Family	(4, 10, 16, 18, 20, 22, 27, 33–35, 38–40, 42, 46, 47, 53, 54, 60, 62, 63, 67, 75, 76, 91–95, 98, 103, 107, 114, 131, 134, 136)
Social support	Family	(79, 81, 94, 96, 98)
Racial/ethnic minority status	Family	(4, 5, 14, 15, 20, 32, 40, 42, 48, 62, 67–69, 76, 96, 106, 108, 114, 134, 141, 142)
Availability of dental insurance	Family	(14, 43, 44, 93, 96, 97)
Access to transportation	Family	(43, 54)
Parental dental anxiety	Family	(96)
Cultural health values	Family	(27, 31, 43, 54, 67, 96)
Supply of dentists	Community	–
Community water fluoridation	Community	(6, 10, 14, 16, 31, 43, 47, 51, 54–58, 64, 73, 97, 102, 106–109, 117, 134)
Public health education	Community	(43, 97, 110–112)

Grounded in the attribution and social learning theories, studies showed that psychological factors such as self-esteem can reduce dental caries experience both through improving brushing with fluoride toothpaste and use of fluoride rinse (143, 144) (Reinforcing loop R1 in Figure 6) and reducing school absence and barriers to learning (Reinforcing loop R2). Self-esteem and oral health quality of life affects school performance and absenteeism and health literacy (145). Higher health literacy increases the chance of eating a healthy diet, which improves the balance between tooth mineral and biofilm and reduces dental caries experiences (54) (Reinforcing loop R2 in Figure 6). Higher health literacy also raises self-efficacy, which increases brushing with fluoride toothpaste and use of fluoride rinse and reduces adolescent dental caries (Reinforcing loop R3). Reinforcing loop R4 captures the impact of self-efficacy on consuming a healthy diet and experiencing lower dental caries. Moreover, adolescents with lower self-efficacy are more likely to be influenced by peer pressure and develop bad habits such as smoking which will subsequently increase their chance of dental caries (Reinforcing loop R5 in Figure 6) (81, 146, 147). Note that reinforcing loops can act as a virtuous or vicious cycle. For example, when reinforcing loop R6 acts as a vicious cycle, it exacerbates dental caries through dental caries-related morbidity, which leads to school absence (21, 137, 148) and over time, it may influence health literacy and lead to a poorer diet and oral hygiene, and more dental caries experience (Reinforcing loop R6 in Figure 6) (138).

As shown in Figure 6, based on Berggren's model of dental fear and anxiety (149, 150), experiencing symptoms of dental caries could lead to fear of negative evaluation, which creates anxiety and avoidance or delay of dental care, leading to further deterioration of dental status (Reinforcing loop R7). Use of dental care depends on accessibility of dental care. Family resources, dental insurance, community characteristics, and school programs affect adolescent access to dental care (151–153). Disadvantaged adolescents tend to rely on overwhelmed safety net dental clinics and may therefore experience delays in obtaining care. This leads to progression of caries that could have been avoided or resolved with less invasive and expensive approaches, and further overloads the dental safety net (154) thus leading to lower accessibility of dental care for disadvantaged individuals (Reinforcing loop R8 in Figure 6). Thus, higher dental caries might overburden safety net clinics and lead to delay and higher dental caries experience of disadvantaged adolescents. Adolescents are twice as likely to forego using dental care if their parents had no dental visit in a year (155) (Reinforcing loop R9).

Balancing loops B1–B4 depict counteracting mechanisms that influence dental caries. After experiencing dental caries, a patient may seek preventive dental care including fluoride treatment that improves the balance between tooth mineral and biofilm and reduces the risk of new dental caries (Balancing loop B1 and B2). Use of dental care may also improve family health behavior and lead to more brushing with fluoride toothpaste and use of fluoride rinse (Balancing loop B3), as well as a healthy diet (Balancing loop B4), which will reduce the risk of adolescent dental caries experience (156, 157).

The map includes both endogenous variables (i.e., dynamic individual and environmental characteristics that arise from within the model boundary) and exogenous factors (i.e., characteristics from outside the model boundary). Characteristics such as racial/ethnic minority status, community water fluoridation, parental education are included as exogenous (non-modifiable) factors to the causal loop diagram. Genetic factors (e.g., quality of saliva and developmental defects of enamel and/or dentin) are also included as exogenous influences (Figure 6).

## 4 Discussion

Our study identifies and depicts the reciprocal interactions among multi-level factors and their contribution to adolescents’ dental caries experience using the system dynamics approach. Previous research has extensively examined individual, family, and community level influences on adolescents’ dental caries experience. Fisher-Owens and colleagues applied the ecological approach and provided a comprehensive list of multi-level factors that affect children's oral health, but underscored the difficulty of capturing causality due to the complex interplay of the factors involved (158). Our study takes a first step to hypothesize these reciprocal relationships and provide a systems perspective to enhance understanding about adolescent dental caries and the persistence of dental caries among adolescents. We identified the feedback mechanisms contributing to dental caries in adolescents based on a review of the literature with nine reinforcing and four balancing loops. These feedback loops encompass individual, family and community level factors.

We categorized factors studied in the literature and their reciprocal interactions at the include individual, family, and community level influences. The majority of the 138 articles, 93% (*n* = 128), examined individual level factors, while 59% (*n* = 82) and 41% (*n* = 56) of the studies included the family and community level factors, respectively. The top three influences frequently examined in the individual-level studies besides demographic factors such as age and sex (14–17), are oral hygiene (43, 46), diet (59), and dental problems (18–20). In articles that included family factors, the most frequently studied sub-factors are parents’ education (16, 48, 84, 91, 92), parental health literacy (48, 54, 91), dental visit (47, 48, 66, 68, 69, 91, 99), and family income (27, 92–95). Lastly, in the community-level studies, geographical context (10, 17, 103, 104), community water fluoridation (6, 10, 14, 47, 106–109), and schools’ oral health programs (56, 97, 113–116) are most frequently studied.

The causal loop diagram mapped in Figure 6 provides multiple insights. First, Reinforcing Loop R1 and R3 hypothesize the downward spiral of deterioration in self-esteem, self-efficacy, and dental health. Evidence-based school programs can work as a leverage by increasing health literacy to turn these vicious cycles into virtuous cycles and improve dental health. Second, reinforcing loop R8 hypothesizes the mechanisms underlying the persistence of dental caries among adolescents, particularly for lower socioeconomic groups. Disadvantaged adolescents are likely to receive dental care in overburdened dental safety net clinics and may therefore have to delay treatment, which exacerbates dental caries and requires more intensive and time-consuming treatments, further straining the system. State and local governments can increase use of dental care by expanding dental insurance, school programs, and improving community characteristics. In addition, the federal government can enact policies to increase the supply of dentists and increase access through improved reimbursement for dental care and expansion of the dental safety net. Third, as specified in reinforcing loop R9, family health behavior and adolescents’ use of dental care reinforce each other. Providing dental insurance for adults through Medicaid expansion has spillover or “welcome mat” effects on children's enrollment (159), meaning that children whose parents become eligible for Medicaid expansion are more likely to gain coverage. Thus, it is likely that targeting parents or children can enhance the dental health of the other group. Fourth, the causal loop diagram depicts multiple mechanisms through which dental caries can be affected and can explain heterogeneity in trajectories of adolescent dental caries. In other words, this map can be used to explain why some adolescents with similar demographic and socioeconomic characteristics may experience different trajectories of dental caries.

Our study's findings offer more than insights – they provide a pathway to actionable strategies, notably in the realm of oral health education. Building on the provided insights, we envision an intervention strategy inspired by motivational interviewing, which harmonizes well with our system dynamics approach. This strategy involves presenting individuals with a personalized menu of solutions to address dental caries at different levels. For instance, individuals might commit to regular brushing, families could reduce soda consumption, and communities could advocate for healthier vending machine policies in schools. What sets our approach apart is its ability to embrace the complexity of real-life feedback loops, making interventions more practical and attuned to people's daily realities. The framework developed in this study also provides a rich foundation for future simulation research and contributes a powerful tool for identifying prevention practices and policies to reduce adolescent dental caries.

This study has some limitations that should be acknowledged. First, the literature search was conducted using two platforms, PubMed, and Cochrane, which may exclude relevant studies from other sources. Although these two platforms serve as comprehensive databases for health studies, a productive extension of this work would be to conduct a systematic literature review with additional databases to minimize the likelihood of omitting studies related to adolescent dental caries. Second, the determination of which studies to include was based on scope rather than quality. Extensions of this work may usefully assess the quality of such studies in addition to their scope. Third, our map draws from existing literature, primarily comprised of cross-sectional studies, which can offer association between variables but cannot establish causality. As such, the causal loop diagram developed in this study is considered a hypothesis suitable for further development and testing using a quantitative simulation approach. Implementation of this causal hypothesis in a simulation model for policy analysis is a logical extension of this study in keeping with the system dynamics approach. Such analysis would reveal which feedback mechanisms are most salient under different circumstances and suggest which interventions to reduce dental caries would provide the greatest leverage.

To sum up, previous research on dental caries among adolescents has identified risk factors without accounting for reciprocal relationships among multilevel factors. To address this gap, this study applies the system dynamics method to develop a novel causal loop diagram, or causal map, of dental caries among adolescents. The literature-based causal loop diagram that we develop in this study identifies several reciprocal mechanisms underlying dental caries among adolescents, encompassing individual-, family-, and community-level factors. We use the map of reciprocal mechanisms to consider complex feedback mechanisms affecting adolescent dental caries that arise from these multilevel factors.To conclude, our findings may contribute to a deeper understanding of the multilevel and interconnected factors that shape the persistence of dental caries experience among adolescents and designing comprehensive interventions to reduce them.
