# Supplementary material for: Systems mapping of multilevel factors contributing to dental caries in adolescents
Source: Front Oral Health. 2024 Jan 31;4:1285347. doi: 10.3389/froh.2023.1285347 (PMC10864617; doi:10.3389/froh.2023.1285347)
Supplement: Supplementary file 2 [file Datasheet2.docx]

Supplementary Material

Supplementary Table 1: Number and percentage of references (n=138) that identify factors at different levels.

| **Individual Level**: 128 references (93% of 138) | | | |
| --- | --- | --- | --- |
|  | ***Biological Factors***: 103 references (80% of 128) | | |
|  |  | Demographics (age & sex) | 81 references (79% of 103) |
|  |  | Dental problems | 46 references (45% of 103) |
|  |  | Salivary factors & dental plaque | 24 references (23% of 103) |
|  |  | General health condition | 21 references (20% of 103) |
|  |  | Form & arrangement of tooth | 17 references (17% of 103) |
|  |  | Body composition | 13 references (13% of 103) |
|  | ***Health Behavior Factors***: 91 references (71% of 128) | | |
|  |  | Oral hygiene | 68 references (75% of 91) |
|  |  | Diet | 52 references (57% of 91) |
|  |  | Health literacy & use of dental care | 32 references (35% of 91) |
|  |  | Smoking habits | 13 references (14% of 91) |
|  | ***Psychological Factors***: 37 references (29% of 128) | |  |
|  |  | Perceptions | 30 references (81% of 37) |
|  |  | Stressors | 10 references (27% of 37) |
| **Family Level**: 82 references (59% of 138) | | | |
|  | ***Socioeconomic Factors***: 71 references (87% of 82) | | |
|  |  | Parents' education | 52 references (73% of 71) |
|  |  | Family income & Standard of living index | 39 references (55% of 71) |
|  |  | Access to healthcare & insurance | 11 references (15% of 71) |
|  |  | Parents' employment | 8 references (11% of 71) |
|  |  | Social support | 5 references (7% of 71) |
|  |  | Access to transportation | 2 references (3% of 71) |
|  | ***Family Behavior Factors***: 45 references (55% of 82) | | |
|  |  | Regular dental visit | 38 references (84% of 45) |
|  |  | Parental health literacy & health behavior | 14 references (31% of 45) |
|  |  | Parental dental anxiety & Mother's Sense of Coherence (SoC) | 5 references (11% of 45) |
|  |  | Family support | 5 references (11% of 45) |
|  | ***Demographics Factors***: 31 references (38% of 82) | |  |
|  |  | Racial/ethnic background | 24 references (77% of 31) |
|  |  | Family cohesion | 14 references (45% of 31) |
|  |  | Parents' age | 6 references (19% of 31) |
| **Community Level**: 56 references (41% of 138) | | | |
|  | ***Public Factors***: 46 references (82% of 56) | | |
|  |  | Geographical context | 25 references (54% of 46) |
|  |  | Community water fluoridation | 23 references (50% of 46) |
|  |  | Public health education | 5 references (11% of 46) |
|  |  | Dental insurance policy | 2 references (4% of 46) |
|  |  | Neighborhood social capital | 1 reference (2% of 46) |
|  | ***School Factors***: 25 references (45% of 56) | | |
|  |  | School's oral health program | 11 references (44% of 25) |
|  |  | Type of school | 10 references (40% of 25) |
|  |  | School's food program | 4 references (16% of 25) |
|  |  | Study program | 2 references (8% of 25) |


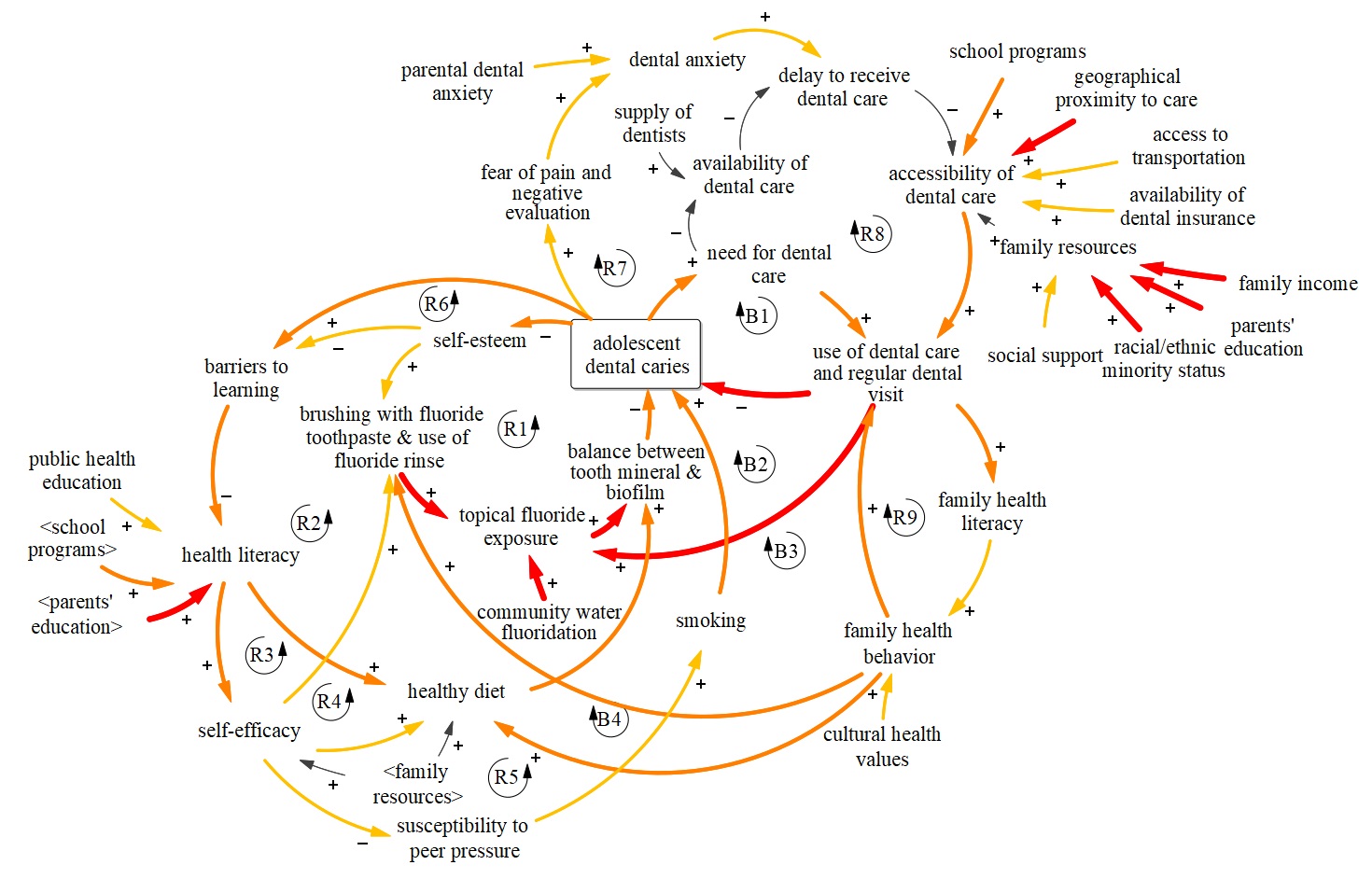


**1 unit of thickness: 2 units of thickness: 3 units of thickness: 4 units of thickness:**

No reference 1-6 references 7-17 references 21-48 references

Supplementary Figure 1: Weighted causal loop diagram of feedback mechanisms affecting adolescent dental caries

Supplementary Table 2: Factors in the causal loop diagram associated with references from the literature

| **Factors in the feedback loops** | **Units of thickness for associated links** | **Number of references** |
| --- | --- | --- |
| **Reinforcing loop R1** | | |
| Self-esteem | 3 | 8 |
| Brushing with fluoride | 4 | 47 |
| Topical fluoride exposure | 4 | 31 |
| Balance between tooth mineral and biofilm (visible plaque) | 3 | 15 |
| **Reinforcing loop R2, R3, R4, R6** |  | |
| Barriers to learning (School performance, absenteeism) | 3 | 8 |
| Health literacy | 3 | 9 |
| Healthy diet | 3 | 16 |
| Self-efficacy | 2 | 5 |
| **Reinforcing loop R5** |  | |
| Susceptibility to peer pressure | 2 | 3 |
| Smoking | 3 | 8 |
| **Reinforcing loop R7** |  | |
| Fear of pain and negative evaluation | 2 | 2 |
| Dental anxiety | 2 | 6 |
| Delay to receive dental care | 1 | 0 |
| Accessibility to dental care | 3 | 7 |
| Use of dental care and Regular dental visit | 4 | 31 |
| **Reinforcing loop R8, Balancing loop B1, B2** |  | |
| Need for dental care (Due to tooth pain) | 3 | 15 |
| Availability of dental care | 1 | 0 |
| **Reinforcing loop R9, Balancing loop B3, B4** |  | |
| Family health literacy | 2 | 6 |
| Family health behavior | 3 | 8 |
| **Exogenous factors** |  | |
| Geographical proximity to care | 4 | 25 |
| School programs | 3 | 14 |
| Parents’ education | 4 | 42 |
| Family income | 4 | 36 |
| Social support | 2 | 5 |
| Racial/ethnic minority status | 4 | 21 |
| Dental insurance | 2 | 6 |
| Access to transportation | 2 | 2 |
| Parental dental anxiety | 2 | 1 |
| Cultural health values | 2 | 6 |
| Supply of dentists | 1 | 0 |
| Community water fluoridation | 4 | 23 |
| Public health education | 2 | 5 |
